# In Vitro Evaluation of the Adsorption Efficacy of Biochar Materials on Aflatoxin B_1_, Ochratoxin A, and Zearalenone

**DOI:** 10.3390/ani13213311

**Published:** 2023-10-25

**Authors:** Michael Appell, Evan C. Wegener, Brajendra K. Sharma, Fred J. Eller, Kervin O. Evans, David L. Compton

**Affiliations:** 1 USDA, Agricultural Research Service, National Center for Agricultural Utilization Research, Mycotoxin Prevention and Applied Microbiology Research, 1815 N. University St., Peoria, IL 61604, USA; 2 USDA, Agricultural Research Service, National Center for Agricultural Utilization Research, Renewable Product Technology Research, 1815 N. University St., Peoria, IL 61604, USA; evan.wegener@usda.gov (E.C.W.); kervin.evans@usda.gov (K.O.E.); david.compton@usda.gov (D.L.C.); 3 USDA, Agricultural Research Service, Eastern Regional Research Center, Sustainable Biofuels and Co-Products Research, 600 E. Mermaid Lane, Wyndmoor, PA 19038, USA; brajendra.sharma@usda.gov; 4 USDA, Agricultural Research Service, National Center for Agricultural Utilization Research, Functional Foods Research, 1815 N. University St., Peoria, IL 61604, USA; fred.eller@usda.gov

**Keywords:** feed safety, biochar, sequestration, animal feed, mycotoxin, aflatoxin, ochratoxin, zearalenone, food safety

## Abstract

**Simple Summary:**

Harmful fungi that contaminate food and feed can negatively impact human and animal health. We measured the ability of safer food-grade and feed-grade materials to remove several mycotoxins that contaminate food and feed. Some *Aspergillus* fungi produce aflatoxins that can contaminate corn, peanuts, and tree nuts. Certain *Aspergillus* and *Penicillium* fungi produce ochratoxin A, which can contaminate cereal grains and fruits. Some *Fusarium* fungi produce zearalenone, which can contaminate corn. Charcoal and biochar materials derived from coconuts and pine tree wood can remove aflatoxin B1, ochratoxin A, and zearalenone under conditions that simulate digestion. These carbon-based materials show promise as greener methods to help reduce exposure to the effects of toxins found in food and feed.

**Abstract:**

Mycotoxin sequestration materials are important tools to reduce mycotoxin illness and enable proper handling of mycotoxin-contaminated commodities. Three food-grade bentonite clays and four generally recognized as safe (GRAS) charcoal/biochar carbon materials that are marketed as feed additives and supplements were evaluated for their ability to sequester the mycotoxins aflatoxin B_1_, ochratoxin A, and zearalenone. The surface area of the clays varied between 32.1 to 51.4 mg^2^/g, and the surface area of the carbon-based materials varied from 1.7 to 1735 mg^2^/g. In vitro, gastric fluid studies indicated that certain pine biochar and activated coconut charcoal could sequester high amounts (85+%) of the mycotoxins at 1 ppm levels or below. However, some biochar materials with lower surface area properties lacked binding capacity. The coconut shell charcoal and pine biochar utilize agricultural waste products in a manner that significantly reduces carbon emissions and provides valuable materials to minimize exposure to toxins found in food and feed.

## 1. Introduction

Various methods are utilized to reduce exposure to natural contaminants in foods, including early detection, prevention, control, and remediation [1]. Recent surveys have found a greater than 70% chance that agricultural commodities can be contaminated with detectable levels of mycotoxins using the most sensitive detection methods [2,3]. New sorbent materials have recently been investigated for their ability to bind or sequester mycotoxins and other natural toxins in fruit juices and other aqueous beverages, fermentations, and during digestion [4,5]. An aqueous environment for binding interactions is critical when using these materials. Several classes of materials have been shown to promote animal health and reduce the effects of mycotoxins in contaminated feed [6]. However, most studies only focus on one mycotoxin, which complicates the evaluation of a given mycotoxin-binding material’s ability to sequester multiple mycotoxins [7]. Multiple mycotoxins have been found in animal feed [8,9]. This study examines the mycotoxin-binding properties of food-grade and feed-grade materials for aflatoxin B_1_, ochratoxin A, and zearalenone.

**Figure 1:**
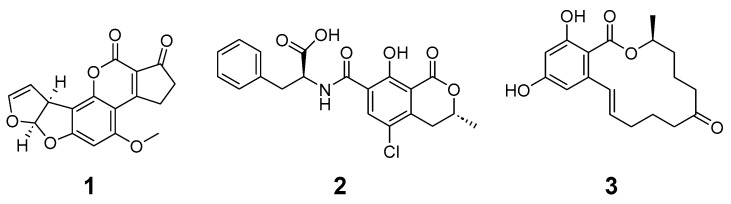
Regulated mycotoxins aflatoxin B_1_ (**1**), ochratoxin A (**2**), and zearalenone (**3**) were investigated in this study.

Aflatoxin B_1_ (**1**) was discovered during the Turkey X disease outbreak in the 1960s in England, in which 100,000 turkeys died suddenly due to high-level liver necrosis and intestinal inflammation (see Figure 1). The source was a nut-based feed contaminated with aflatoxins from *Aspergillus flavus* [10]. Aflatoxin B_1_ is hydrophobic and absorbed through the small intestine and other sites of exposure. Aflatoxin B_1_ rapidly absorbs into the blood stream [1,4,10]. The International Agency for Research on Cancer (IARC) has classified aflatoxin B_1_ as a carcinogen, and its exposure is widely regulated. In the U.S., aflatoxin B_1_ levels are regulated at 20 ppb for corn-related products [11]. 

Ochratoxin A (**2**) is a commonly found mycotoxin in various commodities and is shown to be nephrotoxic, hepatotoxic, teratogenic, and immunotoxic to animals. Ochratoxin A production is most commonly associated with *Penicillium verrucosum* and *Aspergillus ochraceous*; however, several *Aspergillus* and *Penicillium* species are producers, and a wide variety of commodities and commodity-based products are at risk of exposure [12]. Ochratoxin A is absorbed through the gastrointestinal tract and forms a complex with albumin which increases it time of effect [1,12]. Ochratoxin A contains carboxylic acid and phenolic hydroxyl functional groups that can become deprotonated under pH environments associated with digestion. Ochratoxin A exists as monoanionic and dianionic forms under physiological conditions (carboxylic acid pKa at approximately 4.3 and phenolic hydroxyl pKa at approximately 7.2). Over 60 countries have set regulations and regulatory limits varying from 0.100 to 1 ppm in feed products [13]. Reducing ochratoxin A levels from food and feed using chemical-based detoxification is challenging due to its chemical and physical stability. Ochratoxin A is widely regarded as a mycotoxin of concern due to its chemical stability and the wide range of products in which this mycotoxin can occur.

Zearalenone (**3**) is produced by *Fusarium* species that occasionally contaminate agricultural commodities. This estrogenic resorcylic acid lactone derivative possesses a chemical structure similar to 17β-estradiol and acts at sites associated with estrogenic activities. Specifically, zearalenone and its metabolites interact with mammalian estrogen receptors, negatively impacting hormonal regulations. Zearalenone, also known as F2 toxin, is commonly found in wheat and corn [13]. Zearalenone is absorbed through the gastrointestinal tract and can become deprotonated under physiological conditions to exist in anionic forms (pKa of the phenolic hydroxyl is approximately 7.6) [13,14]. Zearalenone is relatively stable, and detection and binding materials are the primary approaches to reducing exposure [14]. Swine are particularly adversely affected by exposure, resulting in reproductive issues detrimental to production.

Recently, several novel approaches have been investigated to reduce exposure to mycotoxins and their metabolites in food and feed, including nutritional supplements. Increasing levels of aflatoxin B_1_ have been reported in Europe with concerns over the formation of toxic metabolites in dairy milk. The effects of natural phytochemical turmeric powder were investigated to reduce levels of the toxic metabolites aflatoxin M_1_ and aflatoxicol in dairy cow milk [15]. The study found that formulations need to be further investigated to improve the bioavailability of turmeric powder. In vitro studies have shown the natural product quercetin can reduce the transformation of aflatoxin B_1_ to more carcinogenic metabolites by increasing production of glutathione [16]. 

Other approaches to remove mycotoxins include a variety of physical and chemical methods, including sorting/separations, washing, heating, irradiation, and chemical treatments [2,17,18]. However, these methods leave decomposition products and adducts, or can be costly for certain commodities. Clays, modified clays, carbohydrates, polymers, surfactants, and absorbing bacteria have been shown to remove levels of certain mycotoxins [2,5,17,18]. The adsorption properties and applications of these mycotoxin-binding materials are known, and there is a need for greener materials with high capacities for a range of important mycotoxins. 

In this study, we investigate the utilization of several commercially available food-grade and feed-grade clay and charcoal materials to bind the regulated mycotoxins aflatoxin B_1_, ochratoxin A, and zearalenone. Biochar is a black, light weight, solid carbon-based form of charcoal that is formed by pyrolysis under oxygen-limited environments [19]. Carbon sequestration is the primary interest of biochar for its ability to reduce carbon emissions by half with minimal efforts. Biochar materials are often marketed as horticulture charcoals and are generally recognized as safe (GRAS) carbonized biomass. Several types of biochar have improved agricultural productivity as soil amendment products through supporting soil aeration, nutrient availability, and water filtration and retention [19,20,21]. The properties of biochar can vary considerably depending on the type of feedstock, temperature of pyrolysis, and any additional chemical/physical steps used to modify the properties of the materials [19]. Recent studies have investigated the use of biochar and other charcoal-related materials to sequester toxins or aid digestion, including reduction of methane production [20]. For centuries, charcoal-related materials have been used to improve health in animal production [21]. With the recent interest in biochar over the past decade, many producers have included biochar in animal feed [22]. The USDA recently released a report on using plant-based activated carbon as a feed additive to reduce exposure to toxins in feed, and several commercially available charcoal and biochar feed additives are of interest for this purpose [6,23]. 

## 2. Materials and Methods

### 2.1. Reagents

Aflatoxin B_1_ (**1**), ochratoxin A (**2**), and zearalenone (**3**), HPLC-grade acetonitrile, phosphoric acid, sodium phosphate, and disodium phosphate were purchased from Sigma-Aldrich Company (St. Louis, MO, USA). Deionized water was used to prepare all reagents (Nanopure II, Sybron/Barnstead). All solvents were HPLC grade. Food-grade calcium montmorillonite was sourced from 5326 Partners, LLC (Sheridan, WY, USA). Sodium montmorillonite was purchased from Spark Naturals (Orem, UT, USA). Food-grade sodium bentonite was purchased from Belle Chemical, LLC (Billings, MT, USA). Food-grade activated charcoal from coconut shells was purchased from NusaPure, Gmax Central, LLC (Orlando, FL, USA). Feed-grade Pine biochar was purchased from High Plains Biochar LLC (Laramie, WY, USA). Horticulture biochar was purchased from Alleo (Bay Minette, AL, USA). Olive wood biochar was purchased from Olivette Atlas Olive Oils (Miami, FL, USA). Chemical structures were created using ChemDraw v22.2.0.3300 (PerkinElmer Software).

### 2.2. Steady-State Fluorescence

A Varian Cary Eclipse instrument (Palo Alto, CA, USA) was used to determine mycotoxins’ excitation and emission wavelengths. Cary Eclipse Scan and Simple Read software v1.1(132) (Palo Alto, CA, USA) were used to record the spectra. A Fisher Scientific Accumet AP71 pH/mV/Temperature Meter (Pittsburgh, PA, USA) was used to determine pH. Steady-state fluorescence spectra were recorded in a 10 × 10 mm quartz cell at room temperature (23–25 °C). The slit widths were set to 5 nm for the entrance and exit slits. A scan rate of 600 nm/min was used for the measurements. Ochratoxin A has an excitation wavelength of 333 nm and an emission of 475 nm. Aflatoxin B_1_ has an excitation wavelength of 365 nm and an emission of 415 nm. Zearalenone has an excitation wavelength of 315 nm and an emission of 460 nm.

### 2.3. Surface Area

Surface area measurements were made using a Quantachrome autosorbIQ automated gas sorption analyzer. Samples were outgassed under dynamic vacuum at 80 °C for 0.5 h, followed by 200 °C for 2 h, and lastly, 300 °C for 4 h. Measurements were performed with N_2_ at 77 K, and surface areas were determined using the BET method over a pressure range of 0.025 < P/P_o_ < 0.20. When necessary, the pressure range used to calculate surface areas was reduced, as described by Rouquerol et al., to account for the filling of micropores [24]. 

### 2.4. FT-IR

The FT-IR experiments were conducted using modifications to previously published procedures [25,26]. The FT-IR spectra were obtained using a Shimadzu FT-IR instrument equipped with an ATR attachment. The apodization was for square triangles. The scans were set at 60. The resolution was set at 4 1/cm. The FT-IR spectra were recorded within the 340–4000 1/cm fingerprint region. 

### 2.5. Equilibrium Sorption Assays

Mycotoxin sorption studies of the clays, charcoals, and biochars were carried out using a Max Q4450 incubating shaker with the rpm set at 60 and the temperature at 37 °C. Various concentrations (0.1 ppm and 1 ppm) of single evaluations of aflatoxin B1, ochratoxin A, and zearalenone were incubated with biochar (1 mg) dispersed in 1 mL solutions (pH 2.0, 100 mM sodium phosphate buffer, and pH 5.5, 100 mM sodium phosphate buffer) in 1.8 mL vials using variations of previously published procedures [5,20,27,28,29,30]. Following the two hours incubation, the samples were centrifuged for ten min at 3800 rpm. Experiments were performed in triplicate. The bound mycotoxin was calculated by subtracting the amount of mycotoxin free in the solution in the presence of the sorbent from standard solutions run without sorbent. The final concentrations were determined using standard curves and LC chromatography.

The percent adsorption of aflatoxin B_1_, ochratoxin A, and zearalenone by sequestering materials was calculated using the following equation:

% Sorption: (Initial Amount − Amount Unbound)/Initial Amount × 100

where the initial concentration is the amount of mycotoxin concentration at the beginning of the experiment (0.1 ppm or 1 ppm) and the amount unbound is the mycotoxin concentration in the supernatant at the end of the experiment. The concentration of the unbound mycotoxin was calculated by comparing the mycotoxin levels with a control group of experiments that did not have mycotoxin-binding materials.

### 2.6. In Vitro Gastric Model Evaluation

The efficacies of the mycotoxin-binding materials were evaluated using a popular gastric model that enabled comparison between other types of materials [5,31,32,33]. A vial (50 mL) containing 2.5 mL of 0.1 M sodium phosphate buffer pH 6.0, 12.5 mg of sequestration agent, and 5 mL of mycotoxin solution (1 ppm in 0.1 M sodium phosphate buffer pH 6.0) was prepared. The pH of the mixture was adjusted to 2.0 using 300 µL of 1 M hydrochloric acid solution (aq) to simulate stomach acid conditions. The 50 mL vials were placed on a Max Q4450 incubating shaker with the rpm set at 60 and the temperature at 39 °C for 2 h. Next, the small intestine was simulated by adding 1 mL of 0.2 M sodium phosphate buffer (pH 6.8) and 300 µL 1 M sodium hydroxide solution. The incubation was continued for an additional 4 h at 39 °C. Following incubation, the solution was centrifuged for 10 min at 3800 rpm. The supernatant was collected to measure the amount of free mycotoxin using LC analysis.

The percent adsorption of aflatoxin B_1_, ochratoxin A, and zearalenone by sequestering materials was calculated using the procedure described in Section 2.6.

### 2.7. LC Analysis

LC analysis was used to quantify levels of aflatoxin B_1_, ochratoxin A, and zearalenone following modifications to previously published procedures [20,29,34,35]. Buffered samples were filtered twice through 0.02 mm PTFE syringe filters, and (100 µL) samples were added to 900 µL of the filtered mobile phase. The LC system included a Shimadzu LC-20AT pump, an RA-10 fluorescence detector (excitation at 365 and emission at 415 for aflatoxin B_1_, excitation at 333 nm; emission at 475 nm for ochratoxin A, and excitation at 315 nm and emission at 460 nm for zearalenone). The system operated with a flow rate of 1 mL/min. The mobile phase for aflatoxin B_1_ was water: methanol (7:3, *v*/*v*). The mobile phase for ochratoxin A levels was acetonitrile/water/acetic acid (495:495:10). The mobile phase for zearalenone, the mobile phase was 1:1 acetonitrile: water. A CBM-20A communication bus controlled the system. A Rheodyne 775 manual injector equipped with a 20 µL loop was used for sample injection. A Phenomenex Luna 5 mm C18 (2) 100A column (250 × 4.6 mm) was used to achieve separation.

### 2.8. Statistics

Sorption binding data are presented as mean of triplicate experiments ± standard deviation values. The variation between samples was analyzed using MicroSoft Excel with the statistical analysis tool and the XLMiner Analysis ToolPak v2.0.0.0 (Frontline Systems). The *p* value was calculated from the T score (www.statology.org, accessed on 18 September 2023). A *p* value of less than 0.05 was determined to be statistically significant for the evaluation of samples.

## 3. Results

### 3.1. Surface Areas of Food-Grade and Feed-Grade Sequestering Agents

The surface areas of the food-grade and feed-grade sequestering agents investigated in this study are provided in Table 1. There is significant variability between the surface areas of charcoal/biochar materials investigated in this study. Calcium montmorillonite (**4a**) had a surface area of 51.4 m^2^/g, which is the higher than the surface areas of the sodium montmorillonite (**4b**) and sodium bentonite (**4c**). The biochar and charcoal samples investigated in this study have a wide range in surface areas from 1.7 to 1735 m^2^/g. 

### 3.2. In Vitro Percent Adsorption of Mycotoxins under Simulated Digestion Conditions

The binding properties of the three clays and four biochar/charcoals for the in vitro gastric model assays are provided in Table 2. In vitro binding of aflatoxin B_1_, ochratoxin A, and zearalenone in simulated digestion experiments was conducted at the 1 ppm level. The simulated digestion model consists of two-hour incubation at pH 2.0 to simulate stomach acid digestion and four-hour incubation at pH 6.8 to simulate the small intestine. Activated coconut charcoal (**5a**) and pine biochar (**5b**) were capable of binding significant percentages of aflatoxin B_1_, ochratoxin A, and zearalenone at the 1 ppm level in the in vitro simulated digestion assay. Olive wood biochar (**5d**) was capable of lowering mycotoxin concentrations significantly, but not as well as activated coconut charcoal (**5a**) and pine biochar (**5b**).

### 3.3. Aflatoxin B_1_ Sorption at pH 2.0

Table 3 shows the sorption studies for aflatoxin B_1_ after two-hour incubation in simulated gastric fluid (pH 2.0) to simulate the effectiveness of the sequestration agents in an environment that simulates the stomach. Activated coconut charcoal (**5a**), pine biochar (**5b**), and olive wood biochar (**5d**) bind greater than 93% of aflatoxin B_1_ at the 0.1 ppm level. Increasing the amount of aflatoxin B_1_ (**1**) to the 1 ppm level reduced the percentage of aflatoxin B_1_ absorbed for all materials evaluated. Calcium montmorillonite (**4a**), sodium montmorillonite (**4b**), sodium bentonite (**4c**), and horticulture biochar (**5c**) bound significantly lower percentages of initial aflatoxin B_1_ at the 1 ppm level compared to activated coconut charcoal (**5a**) and pine biochar (**5b**).

### 3.4. Ochratoxin A Sorption at pH 2.0

Table 4 shows the sorption studies for ochratoxin A after two-hour incubation in simulated gastric fluid (pH 2.0) to study the efficacy of the materials to bind ochratoxin A in the stomach. Activated coconut charcoal (**5a**), pine biochar (**5b**), and olive wood biochar (**5d**) bind greater than 91% of ochratoxin A at the 0.1 ppm level. All of the sequestration agents investigated bound over 61% of ochratoxin A at the 0.1 ppm level. For all materials investigated, the percentage of ochratoxin A bound decreased at the 1 ppm level compared to the 0.1 ppm level. Activated coconut charcoal (**5a**) and pine biochar (**5b**) were able to bind greater than 97% of ochratoxin A at the 1 ppm level after incubation for two hours under conditions that simulate stomach digestion. 

### 3.5. Zearalenone Sorption at pH 2.0

Table 5 shows the sorption studies for zearalenone after two-hour incubation in simulated gastric fluid (pH 2.0). All materials evaluated could bind greater than 46% of zearalenone at the 0.1 ppm and 1 ppm levels under conditions that simulate stomach acid digestion. Increasing the levels of zearalenone from 0.1 ppm to 1 ppm decreased the percentage of zearalenone bound. Activated coconut charcoal (**5a**), pine biochar (**5b**), and olive wood biochar (**5d**) bind greater than 93% of ochratoxin A at the 0.1 ppm level. Activated coconut charcoal (**5a**) and pine biochar (**5b**) could bind over 97% of zearalenone at the 0.1 ppm and 1 ppm levels.

## 4. Discussion

Mycotoxins in the diet cause harmful effects on swine and other animal health and production. In vitro, equilibrium binding assays provide economical screening methods to evaluate toxins’ capacity and affinity, including mycotoxins. In this study, we investigated a wide range of commercially available and generally recognized as safe materials to bind and sequester several mycotoxins of concern in simulated gastric juice and small intestine [32,33]. Seven toxin-binding candidates were investigated, including three bentonite clays and four charcoal products. Montmorillonite is a specific type of bentonite clay. Previous studies have shown that clay and charcoal-related materials bind various mycotoxins well at low concentrations of mycotoxins (0.010 ppm) [5,32,33].

### 4.1. Surface Areas of Food-Grade and Feed-Grade Sequestering Agents

The surface areas of the seven toxin-binding materials are shown in Table 1. The food-grade calcium montmorillonite (**4a**) has 60% more surface area compared to sodium montmorillonite (**4b**) and sodium bentonite (**4c**). Montmorillonite and bentonite are soft clay minerals formed during crystallization from water. Calcium montmorillonite (**4a**) is known to have superior adsorption properties in water and is widely used in cosmetics, detoxification agents, agriculture, and waste treatments [36]. 

Interestingly, the biochar and charcoal-based products vary in surface area from 1.7 mg^2^/g to 1735 mg^2^/g, depending on the feedstock and degree of activation [37]. The activated coconut charcoal (**5a**) possessed micropores with a surface area of 988 m^2^/g, and pine biochar (**5b**) had micropores with a surface area of 463 m^2^/g. Previous studies have shown that the increased temperature of pyrolysis can increase the surface area of biochars/charcoals [38]. The surface area is associated with feedstock and any chemical activation. It has also been demonstrated that surface area is not necessarily related to binding effectiveness [20,39,40].

### 4.2. In Vitro Percent Adsorption of Mycotoxins under Simulated Digestion Conditions

The binding properties of the three clays and four biochar/charcoals are provided in Table 2. In vitro binding of aflatoxin B_1_, ochratoxin A, and zearalenone in simulated digestion experiments were conducted at the 1 ppm level. This toxin level is much higher than previous studies that show near-complete binding of mycotoxins. However, the 1 ppm level is frequently used in animal studies to assess the harmful effects of these mycotoxins in animals [41].

The clay materials (**4a**, **4b**, and **4c**) in this study do not bind most of the three mycotoxins at the 1 ppm level after 2 h of incubation in simulated stomach digestion (pH 2.0) and 4 h in the simulated small intestine (pH 6.8). The charcoals/biochars in this study vary significantly in sorption efficacy. Carbon materials with high surface areas bind most of each mycotoxin at the 1 ppm level (86.7–97.3%). In contrast, horticulture biochar (**5c**) possesses the lowest surface area by BET analysis and binds the lowest amount of each of the mycotoxins of the carbon-based materials evaluated in this study. Activated coconut charcoal (**5a**) and pine biochar (**5b**) show promise to reduce animal exposure to the effects of mycotoxins. The harmful effects extend beyond animal production and have been shown to include contamination of animal milk [42,43]. In addition, charcoals have been shown to be essential components in multi-component binders to aid in reducing the effects of ochratoxin A in broiler breeders [44].

### 4.3. Aflatoxin B_1_, Ochratoxin A, and Zearalenone Sorption at pH 2.0

To gain insight into the binding properties of the sequestration agents for aflatoxin B_1_, ochratoxin A, and zearalenone in the initial breakdown of food during digestion, we conducted a series of sorption experiments under conditions that simulate the stomach. Experiments were conducted with mycotoxin levels of 0.1 ppm and 1 ppm. Table 3 shows the sorption studies for aflatoxin B_1_ after two hours of incubation in simulated gastric fluid (pH 2.0). Interestingly, activated coconut charcoal and pine biochar bind aflatoxin B_1_ at high concentrations of 0.1 ppm and 1 ppm. It is also interesting that olive wood biochar (**5d**) and horticulture biochar (**5c**) bind less. Olive wood biochar (**5d**) binds greater than 90% of the aflatoxin B_1_ at 0.1 ppm but only 63% at the 1 ppm level. Olive wood biochar from pruning waste has been shown to bind other small organic molecules, including promazine and promethazine [45].

Table 4 shows the efficacy of sequestration materials to bind ochratoxin A (**2**) at pH 2.0. All materials exhibit an increase in capacity for ochratoxin A compared to aflatoxin B_1_. Activated coconut charcoal and pine biochar demonstrate high binding capabilities at up to 1 ppm for ochratoxin A. Biochars produced from cashew nut shells have been shown to exhibit ochratoxin A-binding properties. However, that published study did not allow comparisons with other types of binding materials [45]. The surface areas of the cashew nutshell biochar were significantly smaller than the pine biochar and activated coconut charcoal in this study (**5b** and **5a**). In addition, several biochar products and clays had improved binding efficacy compared to ground nut shells [46].

Interestingly, the clays (**4a**, **4b**, and **4c**) exhibit similar binding properties for ochratoxin A. These similar binding properties may be due to the complex structure of ochratoxin A and the multiple formations it achieves through intramolecular interactions. In contrast, aflatoxin B_1_ is more rigid and has been shown to accommodate the binding sites of calcium montmorillonite clays.

The sorption studies of the mycotoxin zearalenone (**3**) binding to the sequestration agents at pH 2.0 are shown in Table 5. The sorbents in this study exhibit increased zearalenone binding compared to ochratoxin A and aflatoxin B_1_. Activated coconut charcoal and pine biochar exhibit excellent potential for binding aflatoxin B_1_, ochratoxin A, and zearalenone and may address the need for binding materials that can bind more than one mycotoxin. Several research groups have reported that clays, carbohydrate-based sorbents, and algae biomaterials are limited in the types of mycotoxins that these materials can sequester [2,17,18,47,48].

An FT-IR analysis was conducted to gain insight into the structural features of the mycotoxin-binding features of the sorbents in this study. Specific functional groups, such as amines, exhibit strong molecular recognition capabilities for zearalenone and ochratoxin A [49,50]. Ochratoxin A and zearalenone have weak acid functional groups capable of forming hydrogen bond interactions. The FT-IR spectra of the biochar and charcoal in this study are similar. Notable peaks in both biochars were the 3400–3600 1/cm region O–H stretching peak, the peaks at 1200–1500 1/cm are associated with C–C and C–O vibrations, and the bending peak at 1400–1500 1/cm is related to the CH_2_ group. The peaks near 1500–1700 1/cm may also represent aromatic carbon-carbon alkene groups, common in biochar. 

The FT-IR analysis provide information on the structural features the bentonite clays in this study. The clays in this study are marketed for food-grade applications and cosmeceutical use, and not necessarily intended to bind the specific mycotoxins evaluated here. Other researchers have reported bentonite clays have exhibited favorable mycotoxin-binding properties [32]. Other studies report that calcium montmorillonite (**4a**) and sodium montmorillonite (**4b**) exhibit characteristic FT-IR Si–O–Si in-plane stretching peaks [50,51], with the sodium montmorillonite (**4b**) Si–O–Si frequency band red-shifted ca. 25 1/cm compared to calcium montmorillonite (**4a**), 1000 1/cm vs. 975 1/cm respectively. Both calcium montmorillonite (**4a**) and sodium montmorillonite (**4b**) show O-H stretching peaks at 3555 1/cm, broad hydration O–H stretching peaks at 3450 1/cm, and H–O–H deformation bands at 1625–1630 1/cm as other researchers have observed in montmorillonite [51]. Sodium montmorillonite (**4b**) exhibited a stronger, 10 1/cm blue shifted Si–O stretching out-of-plane peak compared to calcium montmorillonite (**4a**), 1120 vs. 1110 1/cm, respectively. Characteristic Al–Al–OH bending frequencies for calcium montmorillonite (**4a**) and sodium montmorillonite (**4b**) were observed at 910 1/cm, with the sodium montmorillonite (**4b**) intensity slightly stronger. The Al–Mg–OH bending frequency was observed at 845 1/cm for calcium montmorillonite (**4a**) and sodium montmorillonite (**4b**), which is consistent with results from the literature [52]. The Si–O stretching of quartz/silica was observed for both calcium montmorillonite (**4a**) and sodium montmorillonite (**4b**) at 790 1/cm, with the sodium montmorillonite (**4b**) peaks being slightly weaker [53]. Overall, the cationic effect of Na^+^ vs. Ca^2+^ resulted in relatively little difference in the stretching and bending frequencies of the respective forms of the cationic montmorillonite clays, as was observed previously [54]. 

Sodium bentonite (**4c**) exhibited FT-IR frequency bands similar to those of calcium montmorillonite (**4a**) and sodium montmorillonite (**4b**). An O–H stretching band for sodium bentonite (**4c**) was observed at 3555 1/cm, slightly more intense than the broad O–H hydration band at 3450 1/cm compared to both calcium montmorillonite (**4a**) and sodium montmorillonite (**4b**). Sodium bentonite (**4c**) also exhibited a H–O–H deformation band at 1640 1/cm. An out-of-plane Si–O stretching band was observed for sodium bentonite (**4c**) at 1120 1/cm, similar to the montmorillonite clays. A large Si–O–Si in-plane stretching peak for sodium bentonite (**4c**) at 975 1/cm was blue-shifted ca. 25 1/cm, similar to sodium montmorillonite (**4b**), compared to for sodium bentonite (**4c**). The FT-IR peak at 915 1/cm and the shoulder observed at 850 1/cm were attributed to Al–Al–OH bending and Al–Mg–OH bending in previous studies [54], similar to the cationic montmorillonite clays. The peak observed at 800 1/cm in the sodium bentonite (**4c**) was assigned to the Si–O stretching of quartz/silica, which was blue-shifted ca. 10 1/cm compared to the same peaks for calcium montmorillonite (**4a**) and sodium montmorillonite (**4b**). The peaks observed at ≤ 650 1/cm were attributed to the maximum absorption band of layered aluminosilicates with the shoulder at 625 1/cm and the peak at 510 1/cm assigned to Si–O bending frequencies, similar to those observed by other researchers for calcium montmorillonite (**4a**) and sodium montmorillonite (**4b**) [55,56,57].

### 4.4. Related Applications and Considerations

This study investigates several food/feed-grade and related materials for their ability to sequester three important mycotoxins under conditions that simulate digestion. The utilization of materials that are currently used as feed/food additives has the benefit of being more readily adaptable to real-world applications compared to materials that are not already food/feed-grade additives. Activated coconut charcoal (**5a**) and pine biochar (**5b**) are the most promising materials in this study. The potential non-specific binding of nutrients to carbon-based sequestration agents has led to several recent studies on the effects of charcoals and biochars on animal production to better understand the merits and limitations of the uses of charcoal and biochar as feed additives. Biochar-supplemented feed did not affect the performance of pigs during the two weeks before slaughter [58]. Biochar additives at the 2% levels have been shown to reduce poultry diseases associated with *Gallibacterium anatis* and *campylobacters* [59]. Charcoal and biochar from a variety of sources have been shown to increase feed conversion and increase body weight in broilers and strengthen eggshells, depending on the amount of material included in the feed supply [60,61,62]. Sheep demonstrated increased digestibility of feed that included biochar without an impact on weight gain [63]. Recently, it was shown that activated carbon could bind aflatoxin B_1_, deoxynivalenol, and zearalenone [5]. This study shows that certain food-grade and feed-grade coconut charcoal and pine biochar products can sequester aflatoxin B_1_, ochratoxin A, and zearalenone at 1 ppm levels. These results demonstrate that selected carbon-based materials marketed as feed additives may have the additional benefit of sequestering toxins that occasionally contaminate animal feed. 

## 5. Conclusions

GRAS clays and biochars were evaluated for their ability to bind the important mycotoxins aflatoxin B_1_, ochratoxin A, and zearalenone. The influence of surface award on mycotoxin binding was investigated. It was found that surface area has a significant impact on binding properties. Pine biochar was shown to bind high levels of mycotoxins in gastric fluid during in vitro studies, and the results were comparable to food-grade activated coconut charcoal marketed as a detoxification supplement. Certain biochar-based materials show promise to sequester toxins and may be suitable for further activation to enhance mycotoxin-binding properties. 

## Figures and Tables

**Table 1:** Surface areas of sequestering agents.

	Sequestration Agent	Surface Area (m^2^/g)
**4a**	Calcium Montmorillonite	51.4
**4b**	Sodium Montmorillonite	32.1
**4c**	Sodium Bentonite	32.1
**5a**	Activated Coconut Charcoal	1735
**5b**	Pine Biochar	542
**5c**	Horticulture Biochar	1.7
**5d**	Olive Wood Biochar	17.7

**Table 2:** In vitro percent adsorption of mycotoxins under simulated digestion conditions.

	Sequestration Agent	Aflatoxin B_1_ 1 ppm	Ochratoxin A 1 ppm	Zearalenone 1 ppm
**4a**	Calcium Montmorillonite	28.8 ± 3.7	25.1 ± 2.1	44.2 ± 4.6
**4b**	Sodium Montmorillonite	25.8 ± 4.3	23.0 ± 3.3	33.3 ± 4.8
**4c**	Sodium Bentonite	20.2 ± 4.8	17.1 ± 4.2	33.4 ± 5.8
**5a**	Activated Coconut Charcoal	93.2 ± 1.2	95.9 ± 0.3	88.4 ± 1.2
**5b**	Pine Biochar	93.6 ± 0.7	97.3 ± 0.3	86.7 ± 2.2
**5c**	Horticulture Biochar	36.6 ± 6.2	25.6 ± 1.5	21.8 ± 4.3
**5d**	Olive Wood Biochar	73.4 ± 4.6	81.2 ± 1.9	55.4 ± 2.6

Experiments performed in triplicate. Calculated based on a control that contained no sequestration agents. Percent bound is reported as mean value ± standard deviation.

**Table 3:** Aflatoxin B_1_ (**1**) sorption at pH 2.0.

	Sequestration Agent	% Bound 0.1 ppm	% Bound 1 ppm
**4a**	Calcium Montmorillonite	44.7 ± 1.4	18.5 ± 2.1
**4b**	Sodium Montmorillonite	29.4 ± 1.1	18.8 ± 2.3
**4c**	Sodium Bentonite	31.0 ± 2.3	18.2 ± 1.9
**5a**	Activated Coconut Charcoal	98.7 ± 0.8	85.6 ± 1.0
**5b**	Pine Biochar	99.7 ± 0.5	94.3 ± 0.5
**5c**	Horticulture Biochar	66.8 ± 2.1	16.7 ± 2.2
**5d**	Olive Wood Biochar	93.9 ± 1.4	65.6 ± 4.4

Experiments performed in triplicate. Calculated based on a control that contained no sequestration agents. Percent bound is reported as mean value ± standard deviation.

**Table 4:** Ochratoxin A (**2**) sorption at pH 2.0.

	Sequestration Agent	% Bound 0.1 ppm	% Bound 1 ppm
**4a**	Calcium Montmorillonite	61.0 ± 2.8	39.8 ± 3.1
**4b**	Sodium Montmorillonite	69.6 ± 4.2	38.1 ± 1.3
**4c**	Sodium Bentonite	61.2 ± 2.2	53.5 ± 2.2
**5a**	Activated Coconut Charcoal	99.4 ± 0.5	98.9 ± 0.6
**5b**	Pine Biochar	98.1 ± 1.7	97.8 ± 1.4
**5c**	Horticulture Biochar	74.4 ± 2.6	56.2 ± 4.9
**5d**	Olive Wood Biochar	91.8 ± 2.7	87.3 ± 1.1

Experiments performed in triplicate. Calculated based on a control that contained no sequestration agents. Percent bound is reported as mean value ± standard deviation.

**Table 5:** Zearalenone (**3**) Sorption at pH 2.0.

	Sequestration Agent	% Bound 0.1 ppm	% Bound 1 ppm
**4a**	Calcium Montmorillonite	84.7 ± 1.7	77.3 ± 2.9
**4b**	Sodium Montmorillonite	76.4 ± 1.3	47.0 ± 0.9
**4c**	Sodium Bentonite	74.6 ± 1.4	53.9 ± 1.2
**5a**	Activated Coconut Charcoal	98.5 ± 1.8	97.6 ± 1.1
**5b**	Pine Biochar	99.1 ± 1.7	98.6 ± 1.2
**5c**	Horticulture Biochar	65.9 ± 2.2	46.0 ± 3.4
**5d**	Olive Wood Biochar	93.4 ± 1.5	89.6 ± 2.3

Experiments performed in triplicate. Calculated based on a control that contained no sequestration agents. Percent bound is reported as mean value ± standard deviation.

## Data Availability

The data presented in the study are available.
